# Associations Between Achievement Goal Orientations, Preferred Learning Practices, and Motivational Evaluations of Learning Environment Among Finnish Military Reservists

**DOI:** 10.3389/fpsyg.2022.902478

**Published:** 2022-06-23

**Authors:** Antti-Tuomas Pulkka, Laura Budlong

**Affiliations:** Department of Leadership and Military Pedagogy, Finnish National Defence University, Helsinki, Finland

**Keywords:** goal orientation, motivation, learning environment, self-efficacy, thinking styles

## Abstract

In this study, it was examined whether individuals' self-efficacy, preferred forms in learning, and evaluations of the learning environment vary as a function of their goal orientation profiles. It was also explored whether the preferred forms in learning played a role in this association. The participants were 177 reservists of Finnish Defense Forces participating in rehearsal training exercises. Four homogeneous groups based on goal orientation profiles were found: mastery oriented (*n* = 47, 26.5%), success-performance oriented (*n* = 49, 27.7%), indifferent (*n* = 43, 24.3%), and avoidance oriented (*n* = 38, 21.5%). The mastery-oriented group and the success-performance-oriented group reported higher levels in self-efficacy, legislative form in learning, and mastery goal structure when compared to the avoidance-oriented group or to the indifferent group. The avoidance-oriented group reported elevated levels of perceived strain and performance goal structure in comparison to the mastery-oriented group. Controlling the learners' preferences for different forms in learning revealed some slight differences in the observed pattern of between-group differences regarding perceptions of performance goal structure and self-efficacy. Controlling for the legislative form of learning diminished the difference between the mastery-oriented and the avoidance-oriented groups in perceptions of performance goal structure, and controlling for the executive form of learning revealed differences between success-performance oriented and the indifferent and the avoidance oriented. The role of the learning environment in highlighting certain types of activities in learners' choices and the relevance of this regarding their goal preferences are discussed.

## Introduction

Learners' activities in achievement situations are guided by both individual factors and environmental cues (e.g., Magnusson and Törestad, 1993; Fraser, 1994). These activities manifest in varying forms of engagement, or attitudes or stances toward certain forms of engagement that reflect, then, both generalized personal factors as well as more acute responses to the environment. Research on motivation in learning comprises these viewpoints on both individuals' motivation as well as the ways the learning environment and instruction hold motivational cues (Urdan, 1997). Individual learner's motivation and his/her view on the learning environment are dependent on each other: learners with different kinds of motivational disposition may act and perform differently in achievement situations, but they also interpret instruction through “motivational glasses” (Fraser and Tobin, 1991; Wolters, 2004). What is more, motivation in learning has both generalized and context-specific components (Pintrich, 2003, p. 676) meaning that despite more generic patterns of cognition, emotion, and behavior, certain environments or topics may elicit varying ways of responses or engagement despite more generic motivational disposition. To take this further, learners may, for example, balance between learning and wellbeing goals (Boekaerts and Niemivirta, 2000, p. 427–431), or, more practically, adapt their study strategies based on their interpretation of teacher's demands (Broekkamp and Van Hout-Wolters, 2007).

Research has shown that different types of motivation lead to different kinds of behavioral outcomes and more practical forms of preferences in what comes to engagement, as well as perceptions of instruction (Niemivirta, 2002a; Tapola and Niemivirta, 2008; Pulkka and Niemivirta, 2013). Also, it has been shown that preferences for different styles or forms of learning activities are related to how the learning environment or instruction is perceived (Simpson and Du, 2004; Akkoyunlu and Soylu, 2008). However, to our reading, the interaction of these two effects has been less examined.

What comes to context, we examine these interactions in a special environment of the reserve training exercise in Finnish national defense scheme. The importance of the context is emphasized as military training universally is well-formalized, including, for example, clear instructions, rules, and given orders that are expected to be complied with. Such clear structures might well-highlight the effects of environment on individuals' conduct.

The aim of our study is to examine whether learners' evaluations of their competence and learning environment vary as a function of their motivational profiles, and further explore if varying preferences for learning and studying in a specific environment play an independent role in this.

### Personal Achievement Goal Orientations

Our take on motivation is based on research on achievement goal orientations that are generalized tendencies to value and prefer certain kinds of outcomes in learning and achievement contexts (Urdan, 1997; Pintrich, 2000a, 2003; Elliot, 2005). Early research on achievement goals was based on two somewhat opposing dimensions: task, mastery, or learning goals (goals of personal improving) and ego or performance goals (goal of proving or showing ability) (Nicholls, 1984; Dweck, 1986). Although researchers used different terms to describe the categories. It was postulated that task- or mastery-oriented learners pursue and prefer goals that represent learning new things and gaining competence with intrapersonal reference, whereas performance-oriented learners strive to prove their ability relative to others (e.g., Ames and Archer, 1987; Elliot and Dweck, 1988). The later research has distinguished between approach and avoidance forms of performance goals. In this view, performance-approach goals represent specifically outperforming others and appearing competent, whereas performance-avoidance goals have focus on not appearing less competent than others and avoiding judgements of incompetence. Also, it has been established that learning or mastery can be pursued with varying criteria. It has been suggested, for example, that approach/avoidance-valence applies also to mastery goal pursuit (Elliot and McGregor, 2001) or that the mastery goals can be approached with extrinsic criteria (good grades and other evaluations) (Niemivirta, 2002a).

In this study, we use a five-dimensional model (Niemivirta, 2002a) that includes two mastery goal orientations: mastery-intrinsic orientation that has focus on learning itself and the mastery-extrinsic goal orientation that also focuses on learning but with external criteria, such as grades or other evaluations. Regarding performance-goal preferences, we use the performance-approach and performance-avoidance dimensions. In this conceptualization, it is also postulated that not all learners' strivings refer to achievement or performance. Following this, in this study, we also utilize a dimension of a work-avoidance orientation that reflects aims of minimizing effort and avoiding challenges (Nicholls et al., 1985; Thorkildsen and Nicholls, 1998).

Our analytical strategy is based on the person-oriented approach (see Niemivirta et al., 2019), where the focus is on profiles of scores and their effects instead of associations between variables (Laurse and Hoff, 2006). As an analytical strategy, similar patterns in variables as displayed by individuals are identified and these groups are examined (von Eye and Bogat, 2006). The relevance of the person-centered approach in research on motivational goals arises from the widely accepted multiple-goal perspective, meaning that a person can be motivated by different types of goals simultaneously (e.g., Pintrich, 2003, p. 676; Pastor et al., 2007). Grouping participants based on their scores of multiple goal orientation dimensions aims to reveal the effects of different combinations instead of separate paths between variables.

The results from research on achievement goal orientation profiles indicate that there seems to be somewhat recurring patterns of achievement goal preferences, although studies using this approach differ not only in contexts but also in instrumentation and profiling methods. However, Niemivirta et al. (2019, p. 575–576) present in their review that usually certain categories of profiles seem to emerge (based on pattern of levels in all measured dimensions). These profiles are predominantly mastery goal profile, predominantly performance goal profile, combined mastery and performance goal profile, moderate or low-level profile (on the level of all dimensions), and work-avoidant goal profile (that is, in studies that include work-avoidant dimension) (Niemivirta et al., 2019).

### Classroom Goal Structures

In addition to personal achievement goals, it was postulated by early goal researchers (e.g., Ames, 1992a,b) that this theory also has contextual pedagogical implications. Accordingly, learning environments or instructional features may take forms that hold specific motivational cues. Goal structures represent the motivational classroom climate that is mostly explicated by the teachers, either by the actual instruction or other features (Wolters, 2004; Wolters and Gonzalez, 2008; Bardach et al., 2020). These features emphasize the types of achievement goals on a contextual level; for example, if evaluation of a certain task is based on ranking the students or, in other words, on a comparison between students, it can be argued that this highlights a goal of outperforming others, and thus may foster the adoption of certain achievement goals by the learners (e.g., Ames, 1992a).

The goal structures were first conceptualized by two dimensions. First a classroom that includes mastery-goal structures supports learners to focus on learning and development itself, and understanding of materials, whereas performance-goal structure has a focus on social comparison and demonstration of ability (Midgley and Urdan, 2001; Miller and Murdock, 2007). The performance-goal structure was later on defined to approach and avoidance components: the performance-approach structure includes practices that emphasize outperforming peers and the performance-avoidance goal structure emphasizes avoidance of incompetence, or performing lower than peers (e.g., Midgley et al., 1998, 2000; Karabenick, 2004; Murayama and Elliot, 2009). In more detailed terms of pedagogical recommendation, much of the research concerning aspects of instruction derives from the so-called TARGET framework (Ames and Archer, 1988), which defines six categories of motivationally relevant features: tasks, authority, recognition, grouping, and evaluation. The challenge and diversity of learning tasks have an influence on motivation and learning skills. Authority refers to students' involvement in and responsibility for their learning in terms of available choices in method and pace. Recognition is the use of rewards and incentives in different forms, and grouping means cooperation and peer interaction in groups. Evaluation concerns the practices, standards, and references of evaluation and feedback; and time means the workload and pace in reference to individual differences in knowledge and skills (Ames, 1992a).

### Outcomes and Correlates

Personal achievement goal orientations have distinct outcomes in terms of other motivational factors, affect, and learning (Elliot, 2005; Dweck and Grant, 2008). In brief, mastery orientations usually have more positive correlates than performance orientations. Especially performance-avoidance orientation and work-avoidance orientation have generally maladaptive outcomes (Urdan, 1997; Hulleman et al., 2010).

The mastery goal emphasis predicts positively self-esteem and self-regulation (Middleton and Midgley, 1997), self-regulated and deep or interest-based learning and studying (Senko and Miles, 2008; Yeh et al., 2019), and interest (Harackiewicz et al., 2000). The mastery-extrinsic orientation has shown to be associated with positive outcomes such as commitment and high effort, but it also has links with increased stress and exhaustion (Tuominen-Soini et al., 2008, 2011). The performance-approach orientation has a more mixed pattern of outcomes as, for example, it has been negatively associated with interest-based studying (Senko and Miles, 2008), but positively associated with self-efficacy (Skaalvik, 1997). The performance-avoidance orientation has negatively predicted self-efficacy (Skaalvik, 1997) and self-esteem (Elliot and Sheldon, 1997), as well as interest and enjoyment of lectures (Harackiewicz et al., 2002). The work-avoidance orientation has been shown to have maladaptive consequences and correlates, such as surface-level learning strategies (Ng, 2009) and low interest (Barron and Harackiewicz, 2003).

In sum, when it comes to student evaluations of learning and studying (e.g. interest, enjoyment, competence beliefs, studying preferences), mastery orientation has positive outcomes, performance-approach orientation has mixed outcomes, and the avoidance-focused orientations have negative outcomes.

Different profiles also have different outcomes, and it seems that dominant mastery goal profile and combined mastery and performance-approach goal profile are beneficial in what comes to their correlates and consequences in many respects, such as other motivational factors, wellbeing, and perceptions of learning environment (Niemivirta et al., 2019, p. 577–585).

Mastery-oriented and mastery-performance-approach-oriented students have reported more frequent use of adaptive approaches to learning and tasks (e.g., elaboration, regulation, deep, or analytical approach) and have been more persistent and active, and invested more effort (Valle et al., 2003; Kolic-Vehovec et al., 2008; Pulkka and Niemivirta, 2013, 2015). In comparison, performance and work-avoidance-oriented learners had lower levels of these aspects of learner engagement. However, more mixed results have also been reported: for example, Luo et al. (2011) reported that mastery- performance-approach-oriented and dominantly performance-oriented students reported equally high levels of class, homework, and time management, and high meta-cognitive and effort regulation, when compared to a moderate- or a low-level profile.

Learners with different motivational profiles also differ in their perceptions and preferences of learning environment. Mastery- and/or combined mastery-performance-oriented learners have given more positive evaluations of teaching and assessment methods, clarity of goals, and workload (Cano and Berben, 2009; Pulkka and Niemivirta, 2013, 2015) and have perceived learning environment to be more learning focused, cooperative, meaningful, and include more task variety (Tapola and Niemivirta, 2008; Koul et al., 2012) when compared to learners with other kinds of profiles. Differences that reflect the achievement goal orientation profiles also concern preferences: performance-oriented students have preferred public evaluation practices, whereas avoidance-oriented learners reported less preferences for challenges and task focus in class (Tapola and Niemivirta, 2008).

What comes to relationships between personal motivational orientations and experiences of learning environment, individually varying needs affect the view individual has on the instruction in terms of person-environment match (e.g., Fraser and Rentoul, 1980). The view adopted in this study thus postulates not only that the environment does influence motivational goal preferences but also that learners perceive and interpret a learning environment and instruction in ways (to a certain extent) as a function of their motivational mindset (Fraser and Tobin, 1991; Wolters, 2004; Lyke and Kelaher Young, 2006; Tapola and Niemivirta, 2008; Pulkka and Niemivirta, 2013).

### Self-Efficacy; Believing in Yourself Matters

In addition to personal goal preferences, we also look at students' beliefs of their competence that is importantly associated with learning and motivation, for instance, in a performance context (Zimmerman, 2000). The self-efficacy refers to a learner's personal, often situational, cognitive judgement as an evaluation or a personal belief on how one is able to perform different tasks (Bandura, 1993, 2010; Pajares, 1996; Bong and Clark, 1999). A sense of self-efficacy can be related to what kind of attitude a person has toward challenges and how he/she is dealing with them (Zimmerman, 2000; Pajares and Schunk, 2001).

A high sense of self-efficacy is expected to increase an individual's resilience to work harder and longer even in challenging situations. In case of a mistake or a failure, high reliance on one's competence would make it more tolerable (Pajares and Schunk, 2001). Then again, in the long run, a series of failures undermines a sense of self-efficacy (Bong and Skaalvik, 2003). In addition, a low sense of self-efficacy can even promote avoiding the task at hand (Schunk, 1991).

Interactions between self-efficiency, motivation, and learning can be considered slightly complex. In the context of learning, self-efficiency can vary based on the personal understanding of one's skills, abilities, and past experiences (Zimmerman, 2000; Pajares, 2003). However, it seems that although the results may vary to some extent, mastery- and performance-approach goals predict self-efficacy, but performance-avoidance goals predict self-efficacy negatively (Ahn and Bong, 2019, p. 75–76). What comes to results concerning research on goal orientation profiles, predominantly mastery, and combined mastery-performance profiles have been found to be related higher self-efficacy when compared to other kinds of combinations of personal achievement goal orientation (Luo et al., 2011; Korpershoek et al., 2015).

### Preferred Forms in Learning, Revisiting Thinking Styles

Processes of self-regulation in learning, such as learning strategies, are positively related to students' sense of self-efficacy and motivation (Zimmerman and Martinez-Pons, 1990; Zimmerman, 2000). It follows that individuals differ in their tendencies to evaluate or choose tasks based on the preferred forms of engagement in learning. This is rather an individual's generalized feature than a trait that leads to choosing certain types of activities to perform a task.

Regarding engagement in learning, we rationalize our take on different types or learner activities based on different types of thinking.^1^ In other words, we postulate that different approaches learners choose or would prefer in learning activities arise from their cognitive styles or mindset.

According to Sternberg (1997), one can speak of an individual's style profile or personality-based styles rather than individual ways of thinking. In this theory, a model of cognitive styles consists of five dimensions (functions, forms, levels, scopes, and leanings) that include 13 thinking styles. In our study, we use preferred thinking styles that belong to the dimension of functions. This dimension consists of three different thinking styles: judicial, legislative, and executive (Sternberg, 1990, 1994; Sternberg et al., 2008; Minbashian et al., 2019).

In the particular context of the military environment where essentially the following orders and instructions are emphasized, but on the other hand, initiative is valued. Based on this, we chose to include two classes of thinking styles that specifically refer to these two aspects: the executive and legislative that we hereafter refer to as preferences for forms in learning or engagement.

Individuals with a legislative mindset tend to seek solutions to problems, set their own rules, and be creative. Regarding the executive mindset, the tendency is to do things in familiar ways and face pre-defined problems with precise rules (Sternberg, 1994, 1997).

According to Sternberg et al. (2008), it is natural for individuals with legislative preferences to plan ideas, and they prefer that they themselves can decide what to do and how. More specifically, the legislative form in learning involves independent experimentation, exploring, responsibility, and independence (Sternberg, 1988, p. 202–203).

In turn, individuals with executive preferences prefer for instance tasks that include a clear structure, procedures, or rules, thus emphasizing implementation instead of planning. It involves following instructions, clear instructions, precise boundary conditions, and completing well-defined tasks (Sternberg, 1988, p. 203–204; Sternberg et al., 2008).

Legislative and executive forms in learning may not necessarily be mutually exclusive, but an individual may generally have stronger emphasis on one or the other when performing tasks (Sternberg, 1988, p. 204).

Regarding motivation, learning/mastery orientation has been found to be positively related to legislative preferences among other aspects; in turn, performance-prove orientation was positively related to executive preferences (Minbashian et al., 2019).^2^

Learning preferences have also shown to contribute to academic achievement and they are also related to self-esteem and students' characteristics. For instance, legislative preferences of learning are accentuated with students who are from higher socio-economic-status families and students have reported more extracurricular experience. Finally, executive preferences of learning are related to fewer extracurricular experiences (Zhang and Sachs, 1997; Zhang, 1999).

What comes to associations between learning styles and students' evaluations of instruction, Akkoyunlu and Soylu (2008) examined students' perceptions in a blended learning environment based on different learning styles and showed that students with a preference for logic, thinking, and watching had more positive view when compared to those that prefer observing instead of action. On the other hand, Simpson and Du (2004) found that in an online learning where several types of activities were expected, a preference for logic, thinking, and watching was related to lower level of enjoyment than styles that preferred actively doing things. Despite that these examples, prior studies used different conceptualisations, and that the findings seem to vary; it seems that, in general, student preferences seem to have influence on how they perceive learning environment to some extent.

### The Present Study

For the most, goal structures are operationalised as student measures, in which case students' interpretations of the goals emphasized by the instruction are assessed (Maehr and Midgley, 1991; Lüftenegger et al., 2017). Also, as reviewed above, the student perceptions of instruction are then again slightly affected not only by their motivational mindset in what comes to their preferred goals but also possibly by what kind of activities they prefer and how these preferences match with the pedagogical delivery (Simpson and Du, 2004; Tapola and Niemivirta, 2008). Moreover, learners may hold to some extent varying goal emphasis in what comes to different contexts or domains (e.g., Bong, 2001; Sparfeldt et al., 2015), but less is known whether preferred types of activities or one's stance to different types of work or tasks in achievement situations are more generalized or dependent on domains. Based on this, we consider that by including both these factors (motivational goals and learning preferences) in our analysis, we will be able to highlight the interplay of motivational goals and specific preferences of learning activities in experiencing the learning environment.

In this study, we examine how different motivational profiles (achievement goal orientations) explain the differences in self-efficacy and learners' evaluations of instruction (classroom goal structures). In addition, we examined if thinking styles as forms of preferred engagement play a role in this association.

## Materials and Methods

### Sample

Our sample came from the two army reserve exercises of Finnish Defense Forces and consisted of 177 male soldiers (aged 21–35 years, mean age 23.5) who had filled complete data in the questionnaire. The Finnish reservists are called to rehearsal training most often ~5 years after their national military service that is obligatory for male Finns and voluntary for female Finns. Reserve training is also mandatory and absence requires justified plea; usually a quite high percentage of called reservists take part in exercises.

At the end of the exercise, the participants completed a questionnaire assessing personal achievement goal orientations, preferred learning activity types, and evaluations of exercise's goal structure. The questionnaire was administered by the first author, participation was voluntary, and the participants were assured of the anonymity of measures. The research was approved by the National Defense University as well as the commanding staff of the individual exercises. No personal or sensitive information was collected.

### Instruments

We assessed five types of achievement goal orientations (Niemivirta, 2002a): mastery-intrinsic orientation (two items, e.g., “*To acquire new knowledge was an important goal for me in this exercise*”), mastery-extrinsic orientation (two items, e.g. “*Getting good evaluations was important for me in this exercise*”), performance-approach orientation (two items, e.g., “*An important goal for me in this exercise was to do better than other reservists*”), performance-avoidance orientation (two items, e.g., “*It was important for me not to fail in front of other reservists*”), and work-avoidance orientation (two items, e.g., “*I tried to get away with as little effort as possible in this exercise*”). On these, and all the following scales, the participants rated each statement on a 7-point Likert scale (1 = not true at all, 7 = very true).

The instrument has been used in several studies showing high reliability and validity (e.g., Tuominen-Soini et al., 2011; Pulkka and Niemivirta, 2013, 2015; Tuominen et al., 2020). Confirmatory factor analysis (as implemented in Mplus) was used to verify the structural validity of an instrument. We used the chi-square statistics, the comparative fit index (CFI, cutoff value >0.95), and the root mean square error of approximation (RMSEA, cut-off value <0.06) to evaluate the model fit (cf. two index strategy, Hu and Bentler, 1999). The model fits the data very well: $\chi_{\left(25\right)}^{2}$ = 27.43, *p* = 0.33; CFI = 0.99, RMSEA = 0.023, 90% CI [0.000, 0.066].

For measuring the self-efficacy, we used six items, e.g., “*I can always manage to solve difficult problems if I try hard enough*,” modified from the New General Self-Efficacy Scale (Chen et al., 2001). The NGSE items refer more to complex or challenging situations than to specific knowledge or defined skill, and such a frame of reference is more readily relatable to the military exercise environment, which requires comprehensive adaptation rather than use of one defined skillset. The participants were asked to evaluate the items in reference to their actions in the exercise.

Regarding the self-efficacy scale, the model fits the data reasonably: $\chi_{\left(9\right)}^{2}$ = 33.98, *p* = 0.0001; CFI = 0.95, RMSEA = 0.023, 90% CI [0.000, 0.066] when the error terms of two pairs of variables were specified to correlate.

Features of a learning environment were assessed with three scales. First, we used classroom goal structure scales adapted from PALS (Midgley et al., 2000): mastery goal structure (3 items., e.g., “*My instructor wants us to understand our work, not just memorize it*” and performance goal structure (2 items, e.g., “*My instructor only recognizes really good performance*”). Second, we also measured the perceived (excessive) workload or strain imposed. The rationale to choose this aspect is that the level of challenge or tasks in relation to available time are motivationally relevant (e.g., TIME dimension in TARGET framework cf. Ames, 1992a) and because in practice this is contextually very much salient given the intensive tempo of military exercises. The perceived strain was assessed with two items (e.g., “*My instructors demand too much from us”*). The model of the learning environment scales fits the data well: $\chi_{\left(11\right)}^{2}$ = 15.77, *p* = 0.1499; CFI = 0.98, RMSEA = 0.050, 90% CI [0.000, 0.101]. However, because the internal consistency of performance goal structure was quite low, we chose to use only 1 item that taps well the core of the performance strivings.

Next, as we considered the dimensions of preferred forms in learning new or modified to some extent, at least in this context, we used an exploratory factor analysis to examine the structural features of these scales.

We assessed the preferred forms of engagement with two scales (Niemivirta, 2002b; personal communication November 21, 2021): executive form (2 items, e.g., “*I would like to follow certain rules or instructions in the tasks of the exercise*”) and legislative form (2 items, e.g., “*I would like to experiment new ways of performing tasks and solving problems in the exercise*”). The participants were asked to consider what they think they would like to do in future exercises given their past experience.

Regarding the preferred forms of engagement in learning, the extracted factor solution consisted of two factors with eigenvalue > 1, where the factors explained 60.453% of the variance, and factor loadings were between 0.713 and 0.858. The factors included items that had primary loadings corresponding to the proposed original dimensions (see Appendix 1).

Altogether, based on structural analysis, the composite variables were calculated with the respective internal consistencies (Cronbach's alpha): mastery-intrinsic orientation (α = 0.89), mastery-extrinsic orientations (α = 0.76), performance-approach orientation (α = 0.52), performance-avoidance orientation (α = 0.71), work-avoidance orientation (α = 0.81), self-efficacy (6 items; α = 0.89), legislative form of engagement in learning (2 items; α = 0.80), executive form of engagement in learning (2 items; α = 0.67), classroom mastery approach goal structure (3 items; α = 0.79), classroom performance goal structure (1 item), and perceived strain (2 items; α = 0.66).

The descriptive statistics, internal consistencies, and zero-order correlations are reported in Table 1.

**Table 1:** Descriptive statistics, alphas, and zero-order correlations for all scales.

**Scale**	**M**	**SD**	**α**	**1**	**2**	**3**	**4**	**5**	**6**	**7**	**8**	**9**	**10**
1. Mastery-Intrinsic orientation	4.11	1.70	0.893	–									
2. Mastery-Extrinsic orientation	4.56	1.54	0.761	0.686^***^	–								
3. Performance-Approach orientation	4.27	1.28	0.520	0.411^***^	0.609^***^	–							
4. Performance-Avoidance orientation	3.68	1.65	0.712	0.075	0.185^*^	0.296^***^	–						
5. Work-Avoidance orientation	3.05	1.61	0.810	−0.523^***^	−0.524^***^	−0.351^***^	0.140	–					
6. Self-Efficacy	5.31	0.99	0.889	0.302^***^	0.366^***^	0.528^***^	−0.072	−0.326^***^	–				
7. Legislative form of engagement in learning	4.82	1.34	0.801	0.387^***^	0.413^***^	0.394^***^	0.110	−0.293^***^	0.413^***^	–			
8. Executive form of engagement in learning	3.94	1.24	0.666	0.117	0.121	0.094	0.274^***^	0.175^*^	−0.016	0.089	–		
9. Mastery goal structure	4.90	1.21	0.790	0.445^***^	0.458^***^	0.390^***^	0.077	−0.365^***^	0.369^***^	0.089	0.149^*^	–	
10. Performance goal structure	2.61	1.30	–^a^	−0.107	−0.126	−0.052	0.064	0.199^**^	−0.138	−0.182^*^	0.083	−0.035	–
11.Perceived strain	2.29	1.38	0.655	−0.352^***^	−0.385^***^	−0.250^***^	0.129	0.455^***^	−0.215^**^	−0.196^**^	−0.020	−0.415^***^	0.356^**^

* *p < 0.05*,

** *p < 0.01*,

*** *p < 0.001*.

a *Single item*.

## Results

### Achievement-Goal-Orientation Profiles

A TwoStep Cluster analysis was used to identify homogeneous groups based on the participants' achievement-goal-orientation profiles. The BIC criterion suggested a 3 cluster solution to be the best option (see Table 2). However, regarding the 4-cluster solution, the change in the information criteria was minimal, no exceptionally small clusters were observed, and the correspondence to prior research was clear. Therefore, based on this, we formed four groups following the 4-cluster solution.^3^

**Table 2:** Information criteria values for different clustering solutions.

**Number of clusters**	**BIC**	**BIC change**	**Ratio of distance measures**
1	662.693		
2	549.855	−112.838	2.540
3	536.822	−13.033	1.326
4	539.726	2.903	1.794
5	564.248	24.523	1.078
6	590.745	26.497	1.316

**Figure 1:**
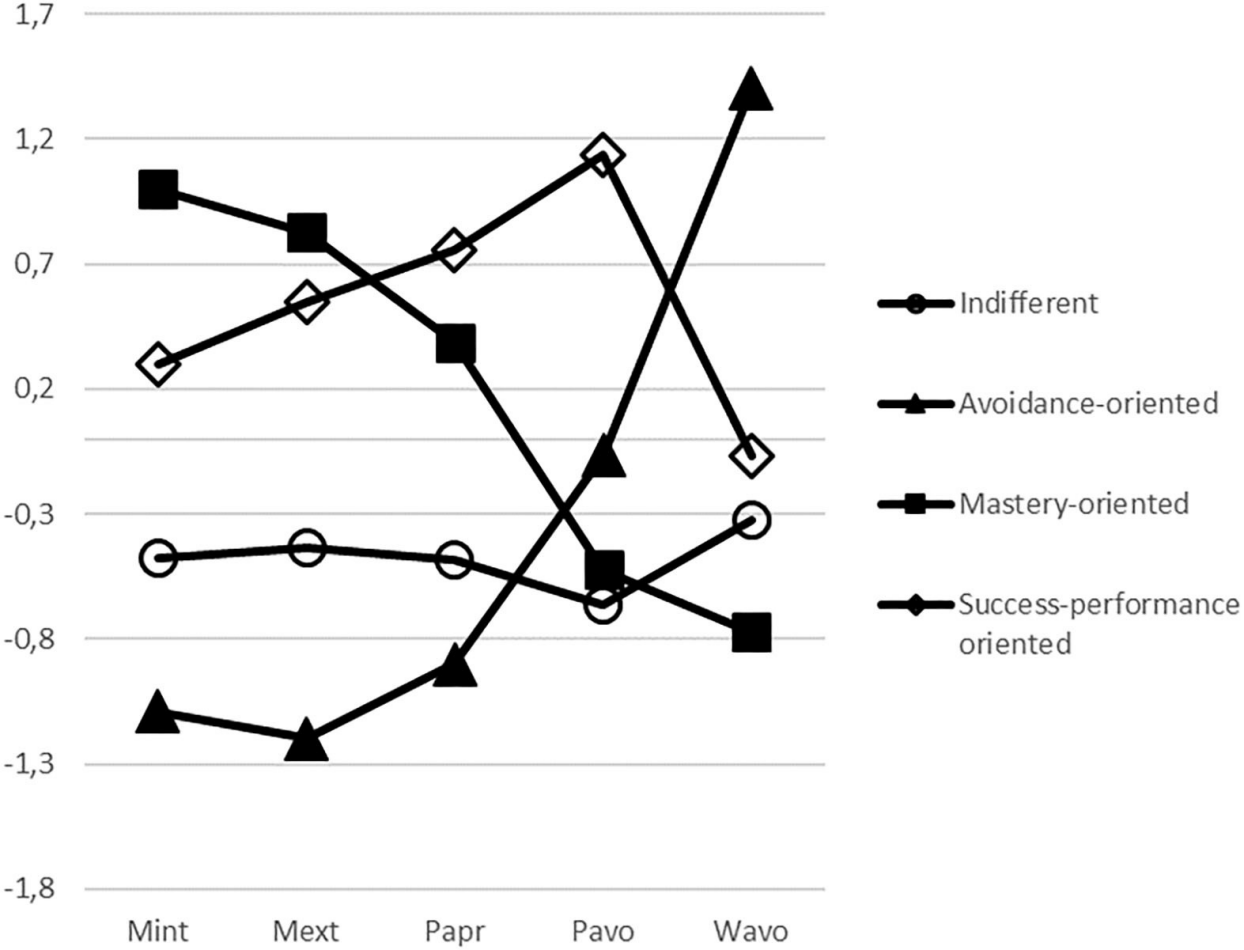
Standardized mean scores on achievement-goal orientation scales as a function of group membership. Mint, mastery-intrinsic orientation; Mext, mastery-extrinsic orientation; Papr, performance-approach orientation; Pavo, performance-avoidance orientation; Wavo, work-avoidance orientation.

Based on the standardized mean score profile (see Figure 1), the group 1 was fairly moderate in all respects without any particular dimension emphasized and labeled indifferent (*n* = 43, 24.3%). The second group scored high on work-avoidance orientation and low on mastery-intrinsic orientation, mastery-extrinsic orientation, and performance-approach orientation in both absolute and relative sense) and was labeled as avoidance oriented (*n* = 38, 21.5%). The third group scored high on mastery-intrinsic orientation and mastery-extrinsic orientation, but low on work-avoidance orientation and performance-avoidance orientation and was labeled mastery oriented (*n* = 47, 26.5%). In the fourth group mastery-intrinsic orientation and mastery-extrinsic orientation were emphasized, and the group scored also high on performance-approach orientation and performance-avoidance orientation, thus indicating focus on both personal success (in intra-individual terms) and display of relative performance (in inter-individual terms). Therefore, the fourth group was named as success-performance oriented (*n* = 49, 27.7%). Mean differences in achievement goal orientations between goal orientation groups are reported in Table 3.

**Table 3:** Mean differences on achievement goal orientations between goal-orientation groups.

**Scale**	**Mastery-oriented**		**Success-performance**		**Indifferent**		**Avoidance-oriented**		***F (df)***	***p***	***η^2^***
	***n*** **=** **47**		**oriented** ***n*** **=** **49**		***n*** **=** **43**		***n*** **=** **38**				
	**M**	**SD**	**M**	**SD**	**M**	**SD**	**M**	**SD**			
Mastery-Intrinsic orientation_1_	5.81^a^	0.900	4.62^a^	1.34	3.30^a^	01.01	2.25^a^	0.992	87.962 (3,173)	<0.001	0.60
Mastery-Extrinsic orientation_1_	5.83_a_	0.951	5.41_b_	0.846	3.90_ab_	0.903	2.72_ab_	01.06	97.860 (3,173)	<0.001	0.63
Performance-Approach orientation_2_	4.76^a^	1.23	5.24^b^	0.778	3.65^ab^	0.961	3.12^ab^	0.866	67.454 (3,173)	<0.001	0.43
Performance-Avoidance orientation^1^	2.81^a^	0.992	5.56^ab^	0.897	2.58^b^	1.17	3.57^ab^	1.48	90.818 (3,173)	<0.001	0.54
Work-Avoidance orientation^2^	1.81^ab^	0.680	2.94^b^	1.14	2.54^a^	0.960	5.32^ab^	1.22	43.355 (3,173)	<0.001	0.61

*Range is 1–7. Group means with the same superscript differ from each other at p < 0.05*.

*Post-hoc test ^1^Tukey HSD, ^2^Games-Howell*.

### Between-Group Differences

The analysis of variance indicated (Table 4) that the goal-orientation groups differed significantly from each other on self-efficacy *F*_(3, 173)_ = 14.867, *p* < 0.01, η^2^ = 0.21, legislative form in learning *F*_(3, 173)_ = 15.144, *p* < 0.001, η^2^ = 0.21, mastery goal structure *F*_(3, 171)_ = 10.944, *p* < 0.001, η^2^ = 16, performance goal structure *F*_(3, 171)_ = 3.226, *p* < 0.05, η^2^ = 0.05, and perceived strain *F*_(3, 171)_ = 13.072, *p* < 0.001, η^2^ = 0.19.

**Table 4:** Mean differences on self-efficacy, preferred forms of engagement in learning, and classroom goal structures between goal-orientation groups.

**Scale**	**Mastery oriented**		**Success-performance**		**Indifferent**		**Avoidance oriented**		***F (df)***	***p***	***η^2^***
	***n*** **=** **47**		**oriented** ***n*** **=** **49**		***n*** **=** **43**		***n*** **=** **38**				
	**M**	**SD**	**M**	**SD**	**M**	**SD**	**M**	**SD**			
Self-Efficacy^2^	5.92^ab^	0.64	5.45^a^	0.71	4.95^b^	1.20	4.77^a^	0.97	14.867 (3.173)	<0.001	0.21
Legislative form of engagement in learning^1^	5.48^ac^	1.02	5.27^b^	1.11	4.31^bc^	1.33	4.01^ab^	1.36	15.144 (3.173)	<0.001	0.21
Executive form of engagement in learning^1^	3.82	1.31	4.25	1.16	3.63	1.2	4.07	1.25	2.217 (3.173)	<0.088	0.04
Mastery goal structure^1^	5.51^ac^	0.98	4.92^b^	1.2	4.70^c^	1.13	4.19^ab^	1.27	10.944 (3.171)	<0.001	0.16
Performance goal structure^1^	2.59^a^	1.90	3.45	1.92	3.35	1.74	3.81^a^	1.91	3.226 (3.171)	<0.024	0.05
Perceived strain^2^	1.66^a^	1.04	2.16^b^	1.20	2.15^c^	1.30	3.34^abc^	1.52	13.072 (3.171)	<0.001	0.19

*Range is 1–7. Group means with the same superscript differ from each other at p < 0.05*.

*Post hoc test ^1^Tukey HSD, ^2^Games-Howell*.

The pairwise comparisons indicated that soldiers with the mastery-oriented profile or the success-performance-oriented profile reported higher scores in self-efficacy, legislative form in learning, and mastery goal structure when compared to the avoidance oriented or the indifferent. The avoidance-oriented group reported higher levels of perceived strain and performance goal structure in comparison to the mastery-oriented group.

### Analysis of Covariance

#### Legislative Form of Engagement in Learning

Series of ANCOVAs were used to find out the association of self-efficacy, evaluations of classroom mastery approach, and evaluations of perceived strain by goal-orientation groups using the legislative form of engagement in learning (called later in the text as legislative form) as a covariate. The Bonferroni pairwise comparisons were used to determine significant differences in the groups (see Table 5).

**Table 5:** Mean differences on self-efficacy, mastery goal structure, performance goal structure, and perceived strain by goal-orientation groups using a legislative form of engagement in learning as a covariate.

**Scale**												**Effect of Legislative form of engagement in learning**		
	**Mastery oriented**		**Success-performance**		**Indifferent**		**Avoidance oriented**							
	***n*** **=** **47**		**oriented** ***n*** **=** **49**		***n*** **=** **43**		***n*** **=** **38**							
	**M**	**SE**	**M**	**SE**	**M**	**SE**	**M**	**SE**	***F (df)***	***p***	***η^2^***	***F (df)***	***p***	***η^2^***
Self-Efficacy	5.79^ab^	0.131	5.37	0.126	5.05^a^	0.134	4.93^b^	0.147	7.169 (3)	<0.001	0.11	1.280 (3)	0.283	0.02
Mastery goal structure	5.57^ab^	0.167	5.14^c^	0.160	4.65^a^	0.175	4.11^bc^	0.188	11.099 (3)	<0.001	0.16	2.607 (3)	0.053	0.05
Performance goal structure	2.71	0.286	3.53	0.272	3.26	0.301	3.65	0.324	2.065 (3)	0.107	0.04	0.065 (3)	0.978	0.01
Perceived strain	1.69^a^	0.191	2.18^b^	0.184	2.13^c^	0.201	3.31^abc^	0.216	10.442 (3)	<0.001	0.16	1.027 (3)	0.382	0.02

*Range is 1–7. Group means with the same superscript differ from each other at p < 0.05*.

Regarding the self-efficacy, the effect of interaction term (goal orientation group x legislative form in learning) was not significant (*F* = 1.280, 3.169, *p* = 0.283), indicating a parallel effect of the legislative form in learning in the profile groups. Significant differences in adjusted means (*F* = 7.169, 3.172, *p* < 0.001) were found between orientation-profile groups even when the legislative form in learning was controlled. The pairwise comparisons indicated that adjusted mean of self-efficacy of the mastery-oriented group (M_adj_ = 5.79, SE = 0.131) was significantly different from the indifferent group (M_adj_ = 5.05, SE = 0.134) and avoidance-oriented group (M_adj_ = 5.37, SE = 0.126) of soldiers. However, avoidance-oriented and indifferent groups did not differ from each other. The legislative form in learning predicts positively self-efficacy.

Regarding the mastery goal structure, the effect of interaction term was not significant [*F* = 2.607 (3.167), *p* =0.053], indicating a parallel effect of the legislative form in learning in the profile groups. Significant differences in adjusted means [*F* = 11.099 (3.170), *p* ≤ 0.001] were found between the orientation-profile groups even when the legislative form in learning was controlled. The pairwise comparisons indicated that the adjusted mean of the mastery-oriented group of soldiers (M_adj_ = 5.57, SE = 0.167) was significantly different from the indifferent group (M_adj_ = 4.65, SE = 0.175) and from the avoidance-oriented group (M_adj_ = 4.11, SE = 0.188) regarding the evaluations of the mastery-goal structure. In addition, the success-performance-oriented group (M_adj_ = 5.14, SE = 0.160) differed from the avoidance-oriented group considering the evaluations of the mastery-goal structure. The legislative form in learning predicts positively the mastery-goal structure.

Regarding the performance-goal structure, the effect of interaction term was not significant [*F* = 0.065 (3.171), *p* = 0.978], indicating a parallel effect of the legislative form in learning in the profile groups. We found no significant differences in adjusted means [*F* = 2.065 (3.169), *p* = 0.107] between the orientation-profile groups.

Regarding the perceived strain, the effect of interaction term was not significant [*F* = 1.027 (3.167), *p* = 0.382], indicating a parallel effect of the legislative form in learning in the profile groups. Significant differences in adjusted means [*F* = 10.442 (3.170), *p* ≤ 0.001] were found between orientation-profile groups even when the legislative form in learning was controlled. The pairwise comparisons indicated that the adjusted mean of the mastery-oriented group (M_adj_ = 1.69, SE = 0.191), the success-performance-orientated group (M_adj_ = 2.18, SE = 0.181), and the indifferent group (M_adj_ = 2.13, SE = 0.201) differed significantly from the avoidance-oriented group (M_adj_ = 3.31, SE = 0.216). The legislative form in learning predicts negatively perceived strain.

#### Executive Form of Engagement in Learning

Series of ANCOVAs were performed similarly, but the executive form of engagement in learning (later called as an executive form) was a covariate in the model instead of the legislative form in learning (see Table 6).

**Table 6:** Mean differences on self-efficacy, mastery goal structure, performance goal structure, and perceived strain by goal-orientation groups using an executive thinking style as a covariate.

**Scale**	**Mastery oriented**		**Success-performance**		**Indifferent**		**Avoidance oriented**					**Effect of executive form of**		
	***n*** **=** **47**		**oriented** ***n*** **=** **49**		**n** **=** **43**		***n*** **=** **38**					**engagement in learning**		
	**M**	**SE**	**M**	**SE**	**M**	**SE**	**M**	**SE**	***F***	***p***	***η^2^***	***F (df)***	***p***	***η^2^***
Self-Efficacy	5.92^a^	0.131	5.46^bc^	0.129	4.94^ab^	0.138	4.77^ac^	0.145	14.801 (3)	<0.001	0.21	0.832 (3)	0.478	0.02
Mastery goal structure	5.53^ab^	0.160	5.06^c^	0.157	4.74^a^	0.172	4.18^bc^	0.177	11.416 (3)	<0.001	0.17	0.489 (3)	0.690	0.01
Performance goal structure	2.59^a^	0.277	3.42	0.270	3.38	0.299	3.79^a^	0.299	3.063 (3)	0.030	0.05	1.178 (3)	0.320	0.00
Perceived strain	1.65^a^	0.185	2.18^b^	0.182	2.13^c^	0.198	3.35^abc^	0.205	13.173 (3)	<0.001	0.19	0.299 (3)	0.826	0.01

*Range is 1–7. Group means with the same superscript differ from each other at p < 0.05*.

Regarding the self-efficacy, the effect of interaction term (goal orientation group x executive form in learning) was not significant [*F* = 0.832 (3.169), *p* = 0.478], indicating a parallel effect of the executive form in learning in the profile groups. Significant differences in adjusted means (*F* = 14.801 (3.172), *p* ≤ 0.001] were found between orientation-profile groups even when the executive form in learning was controlled. The pairwise comparisons indicated that the adjusted mean self-efficacy under the mastery-oriented group (M_adj_ = 5.92, SE = 0.131) and the success-performance group (M_adj_ = 5.46, SE = 0.129) differed significantly from the indifferent group (M_adj_ = 4.49, SE = 0.138) and the avoidance-oriented group (M_adj_ = 4.77, SE = 0.145). The executive form learning predicts positively self-efficacy.

Regarding the mastery goal structure, the effect of interaction term was not significant [*F* = 0.489 (3.167), *p* = 0.690], indicating a parallel effect of the executive form in learning in the profile groups. Significant differences in adjusted means [*F* = 11.416 (3.170), *p* ≤ 0.001] were found between orientation-profile groups even when the executive form in learning was taken into account. When the effect of the executive form in learning was controlled, the effect of orientation-profile groups was still significant. The pairwise comparisons indicated that the adjusted mean of mastery-oriented group of soldiers (M_adj_ = 5.53, SE = 0.160) differs significantly from the indifferent group (M_adj_ = 4.74, SE = 0.172) and from the avoidance-oriented group (M_adj_ = 4.18, SE = 0.177). The success-performance-oriented group (M_adj_ = 5.06, SE = 0.157) differed from the avoidance-oriented group. The executive form in learning predicts positively the mastery goal structure.

Finally, regarding the performance goal structure, the effect of interaction term was not significant [*F* = 1.178 (3.166), *p* = 0.320] that indicates a parallel effect of the executive form learning in the profile groups. Significant differences in adjusted means [*F* = 3.063 (3.169), *p* = 0.030] were found between orientation profile groups even when the executive form in learning was controlled. The pairwise comparisons indicated that the adjusted mean of the mastery-oriented group (M_adj_ = 2.59, SE = 0.277) differed significantly from the avoidance-oriented group (M_adj_ = 3.79, SE = 0.299). The executive form learning predicts negatively classroom mastery structure.

Regarding the perceived strain, the effect of interaction term was not significant [*F* = 0.299 (3.167), *p* = 0.826], indicating a parallel effect of the executive form in learning in the profile groups. The resulting test for equality of the adjusted means found a significant difference [*F* = 13.173 (3.170), *p* ≤ 0.001] in perceived strain between orientation-profile groups even when the executive form learning was taken into account. When the effect of the executive form in learning was controlled, the effect of orientation profile groups is still significant. The pairwise comparisons indicated that the adjusted mean of the mastery-oriented group (M_adj_ = 1.65, SE = 0.185), the success-performance-orientated group (M_adj_ = 2.18, SE = 0.182), and the indifferent group (M_adj_ = 2.13, SE = 0.198) differed significantly from the avoidance-oriented group (M_adj_ = 3.35, SE = 0.205). The executive form in learning predicts negatively perceived strain.

## Discussion

The aim of this study was to examine whether individuals' assessments and beliefs related to their own competence, preferred forms in learning, and evaluations of the learning environment vary as a function of their goal-orientation profiles. It was further explored whether the preferred forms in learning played a separate role in this association.

The goal-orientation-profile groups identified in this study are typical in a sense that they correspond quite well to those found in prior studies, in various age groups, as well as in educational contexts: mastery oriented, i.e. predominantly mastery goal profile; success-performance oriented, i.e., combined mastery and performance-approach goal profile, indifferent, i.e., average- or moderate-goal profile; and avoidance oriented, i.e., avoidant or work-avoidant goal profile (Tuominen-Soini et al., 2011; Niemivirta et al., 2019).

The identified motivational profiles differed in their self-evaluations of competence in a theoretically relevant pattern: mastery focused was related to higher self-efficacy, whereas avoidance focused and/or indifferent profile was maladaptive in this respect. What is more, the success-performance-focused profile was also related to higher self-efficacy, when compared to the avoidance-oriented profile, but not when compared to the indifferent profile, thus indicating that the self-efficacy evaluations in these two groups (success performance/indifferent) were close to one another. This confirms the idea that although the pursuit of performance goals (present in the success profile) may lead to higher achievement (when compared to, for example, mastery focus), this success comes with a price (Harackiewicz et al., 1998; Tuominen-Soini et al., 2008)—in this case, in terms of lower self-efficacy. Lastly, as is suggested also by a prior study (Barron and Harackiewicz, 2003; Ng, 2009), the focus on avoidance forms of performance goals has consistently unfavorable outcomes.

This generic pattern was also confirmed in other aspects. If taken that the legislative form of preferred engagement in learning is the adaptive form in a sense that exercising critical thinking or independent thinking is more desirable than following rules, the mastery-oriented profile appears adaptive. Further, perceiving your learning environment to be promoting understanding and learning—instead of outperforming others or appearing competent—will foster more adaptive motivational outcomes in time, and lastly, as less perceived strain is better than more perceived strain, the pattern described above holds. The mastery focused profile, and—although to lesser extent—success-performance-focused profile are more adaptive than the other two profiles.

However, to take this further, we postulated that perhaps this pattern might partly result also from the person—environment—match, arising from the specific, manneric thinking that the participants have adopted during their prior experience in military training, and may adopt again when returning to this specific educational environment. This, we believe, is indicated by the lack of differences regarding the executive form of preferred engagement. One would expect that the emphasis of legislative form by the mastery- and success-performance oriented should have been mirrored when examining the abiding to rules as in executive form (at least when concerning the mastery oriented—e.g., Senko and Miles, 2008). As this was not observed, it would seem that also those whose motivational disposition fosters preferences of exploration and trying new things also (in the context of military exercise) readily identify the importance and necessity to perform a task as instructed and following set rules.

When taking into account the preference for different types of engagement, we observed both similarities and changes in patterns of between-group differences, that is, when compared between the ANCOVA models and to the results of the series of ANOVAs.

To start with the similarities, the avoidance oriented scored highest in the perceived strain even when the preferred forms were controlled. This indicates that the disposition to strive to avoid effort and challenges is reflected in evaluations of the learning environment in terms of workload and demands by the instructor. Those with strong avoidance tendencies perceive higher strain even independent of their preferences of engagement. Reflecting this to previous studies, it has similarly been found that the avoidance-oriented profile tends to be less adaptive in terms of academic wellbeing and motivation compared to other goal orientation groups (Tuominen-Soini et al., 2012; Tuominen et al., 2020).

Also, the effects of achievement-goal-orientation profiles on the perceptions of mastery-goal structure held regardless of controlling the preferred forms of engagement. Mastery- and success-performance-focused profiles predicted higher perceptions of mastery cues in instruction, when compared with the more maladaptive profiles. Thus, the preferences for different types of instruction and activities do not enter the learners' interpretation of the features in a learning environment that promote learning and development.

Next, regarding the performance-goal structure of the learning environment, the avoidance oriented perceived learning environment to be more performance focused than the mastery oriented, if the executive form was controlled. But if the legislative form was taken into account, this difference was no longer detected. This slight change indicated that the independent effect of preferring looser control or instruction explained partly the perceptions of performance-focused cues in an instruction. We consider this effect to be somewhat small, all in all, but perhaps the preference for legislative form above the other may lessen the sensitivity of learners to interpret their learning environment with terms of social comparison or appearance. However, the results concerning the performance goal structure need to be interpreted with some caution, given that a single-item scale was used in this.

Finally, what comes to the self-efficacy, the results concerning controlling the legislative form were similar to the “baseline” ANOVA pattern, that is the mastery oriented had the most positive self-evaluations when compared to the indifferent and the avoidance oriented. Similar effects have been found in the previous studies (Coutinho and Neuman, 2008). Then, the controlling of the executive form revealed an additional between-group difference; that is, the success-performance oriented now also differed significantly from the groups of a more maladaptive profile. Now, it is quite common that the predominantly mastery and combined mastery-performance profiles are somewhat similar to each other (*cf*. Niemivirta et al., 2019, p. 578), but it seems that, at least in this special context, again, this similarity is slightly affected by what the learners prefer in an instruction. When the preference for rules and strict instruction was controlled, the success-performance oriented appeared to be closer to the mastery oriented in their self-evaluations of their competences.

In summary, our results testify that the associations between personal-goal-orientation profiles and evaluations of learning environments are robust in a way that is only slightly affected by what way individuals prefer to operate in achievement situations. Learners' general and domain-specific achievement goal preferences are known to be somewhat clearly associated (e.g., Sparfeldt et al., 2015; Michel et al., 2020). Also, learners' motivational goal orientations, self-efficacy, and their tendencies in learning activities and metacognition are intertwined (Coutinho and Neuman, 2008; Soyer and Kirikkanat, 2019), which is, in a sense, visible in relationships between achievement goal preferences and self-efficacy beliefs revealed in our study.

Taking this further, the slight differences found do also point out the role of the environment in motivational outcomes (Lyke and Kelaher Young, 1996; Wolters and Gonzalez, 2008). This idea arises from the needs-press model: the personal needs that in our study are represented by tendencies to choose certain goals and prefer certain kinds of forms of engagement, and the learning environment or the environmental press, may the support or frustrate learners' needs, and learners' have a tendency to adapt, to some extent, to the external influence that is the press (Murray, 1962/1938, p. 38–42; Stern, 1970). To clarify, in this study, we do not assume goal orientations to determine preferred forms of engagement in learning or *vice versa* but rather that these factors are in interaction. Certain types of individual preferences are more probable given certain kinds of motivational patterns, but also that the demands of the environment have some influence in this.

Summarizing from the point of view of achievement goal theory, our findings indicate that the motivational profiles identified in this specific context and selective sample correspond well to prior research (for review, see Niemivirta et al., 2019), indicating that the basic principle that goal orientations are somewhat generalized dispositions is valid even in our circumstances or context. Also, regarding multiple-goal perspective, our findings show that the differential effect of certain goal patterns (e.g., Pintrich, 2000b; Linnenbrink and Pintrich, 2001) may be potentially partly explained by preferences for certain types of activities or patterns of behavior the learners acquire through adaptation to environmental pressures. Moreover, as differentially motivated learners' perceptions of instruction were slightly affected by their preferences for engagement, it seems reasonable to argue cautiously that certain types of preferences are more favorable than others, in terms of interplay between personal and classroom goals (Lau and Nie, 2008).

Regarding practical, instructional implications, we suggest that to start with, the educators need to be aware that across contexts and age groups, common motivational variation can be expected, and that pedagogical delivery and one's own competence are interpreted in different ways that relate to these motivational patterns. What is more, individuals prefer different things in learning context: clear guidance and sets of rules may appear restrictive to some learners, whereas others may perceive degrees of freedom in classwork as lack of instruction. However, the learners may adapt their preferences if exposed to a very strict or rigid instructional climate for a length of time. It can safely be assumed that a learning environment that would be optimal to every student is unrealistic, but identifying relevant features in instruction and trying to balance between guidance and exploration with a purpose of scaffolding responsibility and interest in learning is a sound principle supported by our results.

All in all, some limitations are to be taken into account when considering the findings of our study. First, our data was cross-sectional, so the main effects are not to be taken as evidence of causality as such. Second, the exercises in which we gathered data were relatively short, so the actual dynamics of how and with what mechanism the participants preferences were formed, or in other words, what was the specific influence of the environment, remain to be examined in future studies. Lastly, we also do not have in our data measures to represent actually how the instruction was delivered, but this was only assumed based on general information and first-hand experience from other exercises. Hence, we have no direct information of how the role of the instructors may have varied within or during the training, in terms of authoritative role instructors took, or how direct they were in what comes to interaction with trainees. We recommend that these effects should be studied in the future with longitudinal data and specific measures of the forms of instruction, or perhaps by observing the pedagogical delivery in a field.

To conclude, due to the specific sample and context, we do not suggest that these findings are generalisable to different contexts. Rather, we present that motivational profiles in this selective sample and in a very special context were similar to those observed in more generic environments and populations, and their theoretically relevant main effects were also extended to our context.

## Data Availability Statement

The datasets presented in this article are not readily available because authors are not entitled to share data concerning military personnel without specific application that would be duely processed—according to the instruction at the moment. National Defense University is piloting open data procedures during 2022. Requests to access the datasets should be directed to antti-tuomas.pulkka@mil.fi.

## Ethics Statement

This study was reviewed and approved by National Defense University, Finland. Written informed consent for participation was not required for this study in accordance with the national legislation and the institutional requirements.

## Author Contributions

A-TP was responsible for the designing of the study and planning of the article, as well as collecting the data. LB was responsible for most of the data analyses of the study, but the A-TP commented on the application of analysis and preliminary results. A-TP and LB were responsible for writing of the article, but the A-TP commented on and made suggestions for editing the text as a whole. Both authors contributed to the article and approved the submitted version.

## Conflict of Interest

The authors declare that the research was conducted in the absence of any commercial or financial relationships that could be construed as a potential conflict of interest.

## Publisher's Note

All claims expressed in this article are solely those of the authors and do not necessarily represent those of their affiliated organizations, or those of the publisher, the editors and the reviewers. Any product that may be evaluated in this article, or claim that may be made by its manufacturer, is not guaranteed or endorsed by the publisher.

## Footnotes

^1^Sternberg (1988) used term thinking styles in his theoretical work. Given that learning and thinking are intertwined, we rather use a concept of preferred forms of engagement in learning instead of the thinking styles to highlight the fact that the “styles of thinking” refers more to variance in tendencies of behavior and action rather than fixed categorical styles. Also, the term thinking styles may be mixed by readers to learning style research that includes unwarranted assumptions we do not postulate.

^2^In Minbashian's (2019) study, thinking styles were divided into two. Type I included legislative and judicial thinking styles, liberal leanings (prefer to run tasks or projects in a novel way or unfamiliar way), and hierarchic forms (refers to individuals who prefer to run multiple tasks in a given time frame and with different priorities). Type II included executive thinking style, conservative leaning (prefer running tasks and projects in a traditional and familiar way), and monarchic form (prefer to run only one task or project at a time until finished) (Sternberg, 1988; Minbashian et al., 2019).

^3^Despite critique on regular clustering techniques in the past (e.g., Pastor et al., 2007), the two-step cluster analysis has performed equally sufficiently in comparison, for example to latent class cluster analysis (e.g., Benassi et al., 2020) and we deem it an appropriate choice, with sufficient indicators. What is more, when performing a two-step cluster analysis with the SPSS software, it should be noted that the cluster solutions may in some cases appear relatively unstable. In other words, the final solution of the clusters may depend on the order of the cases. To minimize the impact of order, cases can be randomly rearranged. It is recommended to run the cluster analysis again with the SPSS software a few times and obtain different solutions where the cases are sorted in random order (IBM, 2016). According to this stability testing, the cluster solution used in this study was stable.

## Appendix

**Table A1:** Two-factor solution and results of item analysis for preferred forms of engagement in learning.

**Factor/Items**	**α**	**Factor loading**	**Corrected item-total correlation**	**Variance explained**
1. Legislative form of engagement in learning	0.801			34.46
*I would like to experiment new ways of performing tasks and solving problems in the exercise*		0.858	0.671	
*There should be field problems in the exercise that could be solved in ways of one's own choosing*		0.775	0.671	
2. Executive form of engagement in learning	0.666			25.99
*There should be field problems and tasks in the exercise where one can follow a specific routine or given instructions*		0.728	0.499	
*I would like to follow certain rules or instructions in the tasks of the exercise*		0.713	0.499	
